# Delayed presentation of intramural cecal hematoma with challenges in the treatment. A case report and review of the literature

**DOI:** 10.1016/j.ijscr.2021.105884

**Published:** 2021-04-10

**Authors:** Hussam I.A. Alzeerelhouseini, Yousef S. Abuzneid, Osama Y. Aljabarein

**Affiliations:** a Al-Quds University, Faculty of Medicine, Jerusalem, Palestine; b Princess Alia Governmental Hospital, Hebron, Palestine

**Keywords:** ICH, Intramural cecal hematoma, Cecal hematoma, Colon hematoma, Intestinal hematoma, Trauma, Evacuation, Case report

## Abstract

**Introduction:**

Intramural cecal hematomas are rarely encountered clinical entities with only 14 cases reported in our literature. It is usually reported after blunt external trauma, endoscopy-related trauma, coagulopathies, and occasionally spontaneous. Most cases presented acutely after primary insult; however, a delayed presentation rarely can occur.

**Case presentation:**

Herein we describe an 8-year-old male patient who presented to the emergency department with an appendicitis-like picture six weeks after a history of falling. Intraoperatively, the patient was found to have a normal appendix. However, a large intramural cecal hematoma contains black blood with many clots was found. The patient was treated by hematoma evacuation with preservation of the cecum and colon and he was discharged with uneventful recovery. During two years follow up there was no history of complications or recurrence.

**Discussion:**

Intramural cecal hematoma could be presented with various symptoms that range from mild abdominal pain to severe abdominal pain with vomiting, intestinal obstruction, bowel perforation, and hemoperitoneum. Diagnosis is challenging, and CT scan is the key for diagnosis. Although Conservative therapy is the first-line treatment; however, surgery still has a role in selected cases.

**Conclusion:**

The authors report the first case of cecal hematomas with a delayed presentation. The optimal treatment should be individualized according to different etiologies, the patient's stability, and the presence of complications.

## Highlights

• Intramural cecal hematoma is a rare condition with only 14 reported cases.
• It can mimic acute appendicitis.
• A history of trauma, if present, may offer a pointer to the diagnosis.
• Conservative treatment is the first choice in management. However, surgery still has a role.
• Evacuation of cecal hematoma can preserve the colon in selected patients.

## 1 Introduction

Intramural hematoma of the bowel is a rare complication of blunt abdominal trauma caused by tearing of the terminal arterial vessels as they leave the mesentery to penetrate the muscularis layer of the intestinal wall [1]. Although trauma is the most common cause, other causes may include anticoagulant therapy or bleeding diathesis such as hemophilia and leukemia. Rarely did it occur as an iatrogenic consequence, or a rare complication of vaginal delivery [2].

Intramural hematoma may occur at any site from the esophagus to the rectum, being the duodenum is the typical site for it [1,3]. However, Intramural hematoma of the colon is a rare disease with only a few cases have been reported with sigmoid is the most common site [2]. Intramural cecal hematoma (ICH) is extremely rare with only 14 cases reported in our literature.

Most cases of ICH presented in less than 24 h after the primary insult with right hemicolectomy is the treatment if observational therapy was failed [1,[4], [5], [6], [7], [8], [9], [10]]. Herein we report the only case of intramural cecal hematoma with a delayed clinical presentation and the second reported case that treated surgically by hematoma evacuation instead of right hemicolectomy. A review of the Literature has been made, and diagnostic and therapeutic management options are discussed. The work has been reported in line with the SCARE criteria [11].

## 2 Case presentation

An 8-year-old male patient presented to the emergency department after a history of falling from height (3 m). On examination, the patient was well with mild abdominal pain and contusions in the upper and lower extremities. A focused assessment with sonography for trauma (FAST) was negative and serial complete blood counts (CBC) showed no drop in hemoglobin. The abdominal ultrasound performed the following day showed no intraabdominal fluid collections and the abdominal pain disappeared, so he was discharged in a good condition.

Six weeks later, the patient presented to the emergency department complaining of pain in the right lower abdomen for two days’ duration. The pain increased gradually and was associated with fever (38.5 °C), anorexia, nausea, and vomiting; however, he had no trouble in passing stools and flatus. Otherwise, the vital signs were within normal. On examination, the patient looked ill and irritable. The abdomen was flat on shape with localized tenderness in the right iliac fossa. Rebound tenderness in the right lower quadrant was positive. Rigidity and guarding were not found, and examination of the genitalia was normal. The patient and his family don’t have any history of a bleeding disorder, and he is free of medications.

Laboratory investigations showed leukocytosis of 13,000 cells/mm3 with a neutrophil predominance (73 %), normal Hb level (13.8 g/dl), and normal urine analysis. Abdominal ultrasound showed a poorly visualized appendix.

**Fig. 1:**
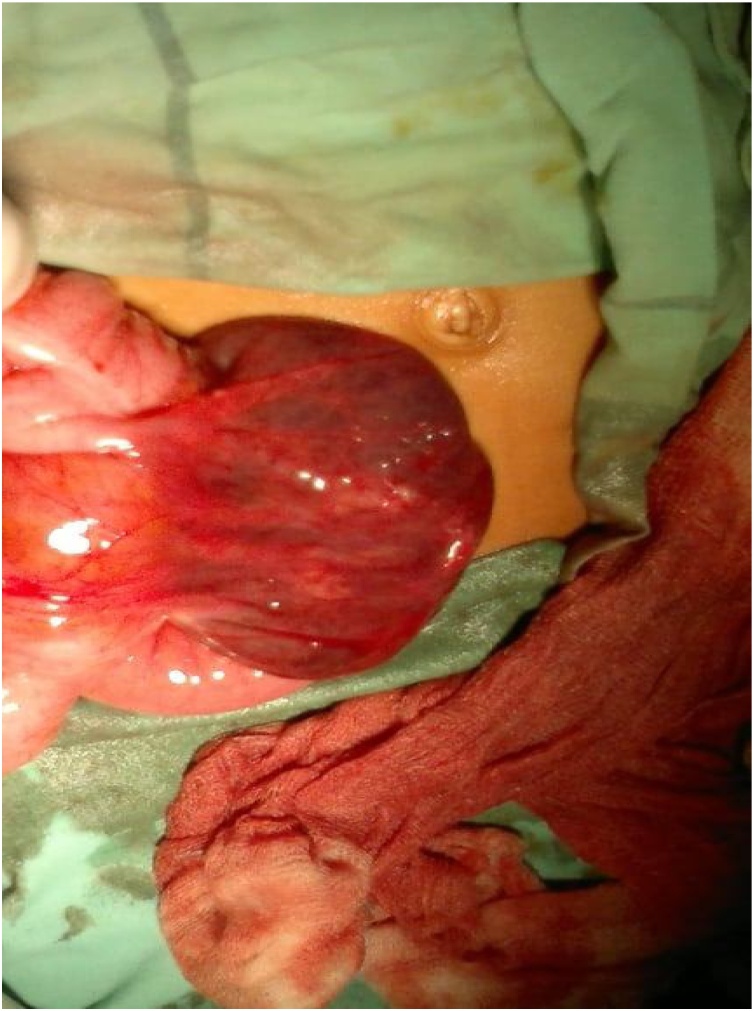
Intraoperative picture showing the subserosal cecal hematoma.

**Fig. 2:**
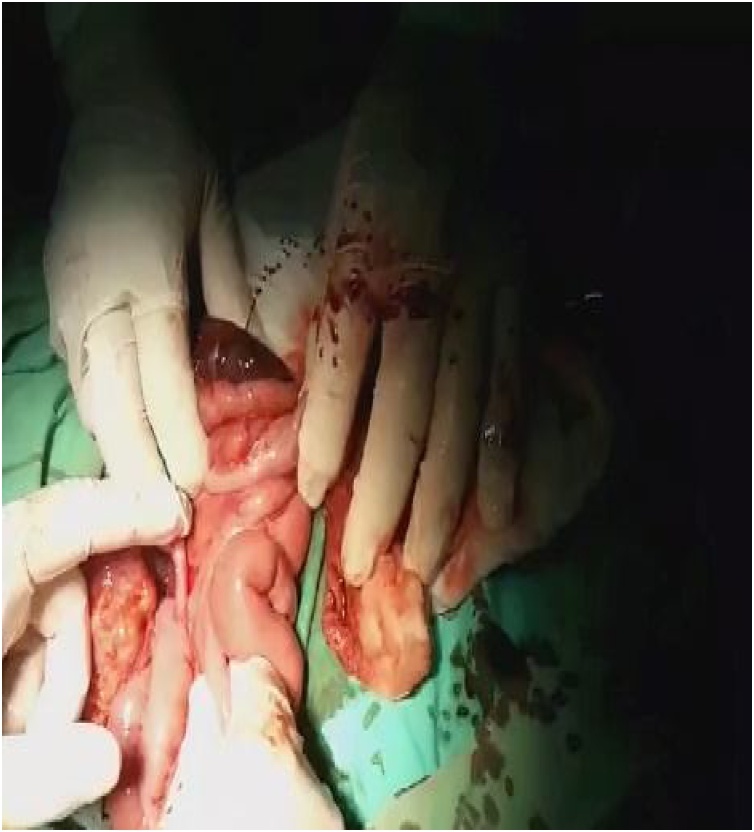
Dark blood released from the cecal hematoma.

At this point, the clinical differential diagnosis was acute appendicitis, and the patient was prepared for urgent appendectomy through a Gridiron incision. Intraoperatively, a subserosal cecal hematoma 10 × 8 cm extending from the antimesenteric to the mesenteric border of the cecum was found (Fig. 1). The hematoma was not expandable or pulsatile and there was no extension to the ascending colon with the appendix grossly appeared normal. Appendectomy was performed and the cecal hematoma was evacuated, which showed dark blood with, many clots (Fig. 2). After ensuring that there was no oozing following the evacuation, interrupted repair of the cecal seromuscular layer by vicryl 3-0 was executed and a pelvic drain was applied.

Postoperatively, the patient was kept NPO for three days; so, intravenous fluid, parenteral ciprofloxacin, and metronidazole were given and also Pethidine when needed. On a postoperative day 2 (POD-2), the patient passed flatus. At POD-3, he was given sips of water and he tolerated them well, so oral intake was started gradually. At POD-5, the patient passed stool and the abdominal ultrasound was normal with no fluid collection. Then, he was discharged with no symptoms. Follow-up for the patient at POD 10 with history, exam, and abdominal ultrasound was normal. The patient had several follow-ups for two years and he was in good condition, without complications or recurrence.

## 3 Discussion

Colonic intramural hematomas are rare [12,13] and they could occur at any segment of the colon [14]. The rectum and sigmoid colon were relatively commonly involved sites [12]. The etiologies may include abdominal trauma, anticoagulant therapy, bleeding diathesis, or iatrogenesis [2]. Hematoma of the colon is much less frequent than hematoma in other segments of the gastrointestinal tract [3]. It can be supposed that it may be due to the protective role of the Teniae coli, which could prevent the diffusion of the hemorrhage in the bowel wall [15]. A review of all published cases of intramural cecal hematoma (ICH) illustrates the variety in clinical presentation, the various etiologic factors, different modalities of treatment, and the outcome of the lesion (Table 1). By analyzing the data of all reported cases (Table 2), trauma was the cause in 8 patients (53 %), colonoscopy was the cause in 3 patients (20 %) who were on anticoagulant thereby. Other less frequent causes were Hemophilia, Subcutaneous Heparin injection, and Spontaneously occurring.

**Table 1:** Summarized clinical data of all published cases of intramural cecal hematoma.

Case	Author, Year	Age	Sex	Etiology	Clinical Manifestations	leukocytosis	Time to presentation	Diagnosis	Size of hematoma (cm)	Hemoperitoneum	Ascending colon extension	Treatment	Complications
1	Bastionelli,1915 [6,26]	26 yr.	M	Struck in abdomen by handle of bicycle	Vomiting, abdominal pain, and distention, intestinal obstruction.	Not reported	Not reported	Intraoperative	Not reported	Yes	Yes	Right hemicolectomy	Not reported
2	Stretton,1920 [6,26]	22 yr.	F	Lifting heavy potted plant	Vomiting, abdominal pain and a mass in the RLQ, intestinal obstruction.	Not reported	Not reported	Intraoperative	Not reported	Yes	Not reported	Hematoma Evacuation	Not reported
3	Nance,1968 [4]	29 yr.	M	Traffic accident	Abdominal pain, intestinal obstruction.	Not reported	Within hours	Intraoperative	Not reported	Yes	Yes	Right hemicolectomy	Not reported
4	Jeffrey,1982 [5]	33 yr.	M	Traffic accident	Midabdominal and RLQ pain	No	Within hours	Abdominal CT scan	Not reported	Yes	Yes	Right hemicolectomy	Not reported
5	Yin,1997 [6]	37 yr.	M	Abdominal trauma (stone)	Abdominal pain, tenderness and guarding in the RLQ, intestinal obstruction.	Yes	Within hours	Abdominal ultrasound and CT scan	10 × 10 × 5	No	Yes	Ileocolic resection	No
6	Calabuig,2002 [1]	21 yr.	M	Falling down on his feet.	Abdominal pain, guarding, and a mass in the RLQ	Yes	24 h	Abdominal CT scan	15 × 8	yes	Yes	Right hemicolectomy	No
7	Calabuig,2002 [1]	33 yr.	M	Abdominal trauma (traffic accident).	Abdominal pain, vomiting, tender mass in the RLQ.	Yes	24 h	Abdominal CT scan	12 × 9	yes	yes	Right hemicolectomy	No
8	Gallo,2003 [7]	69 yr.	F	Colonoscopy.	RLQ pain	No	24 h	Abdominal CT scan	9 × 10 × 15	Not reported	Yes	Observation	No
9	Nakayama,2006 [27]	65 yr.	M	Hemophilia A.	Abdominal pain, bloody stool, RLQ mass, intestinal obstruction, pallor	No	–	Intraoperative	5	No	No	Right colectomy	Gastrointestinal tract bleeding.
10	Jongwutiwes,2008 [8]	74 yr.	M	Colonoscopy.	Abdominal pain and tenderness, RLQ mass	Yes	6 h	Abdominal CT scan	10 × 16	Not reported	Yes	Observation	No
11	Koczka,2009 [9]	86 yr.	M	Colonoscopy.	Asymptomatic	No	Immediately	Colonoscopy	3	No	No	Observation	No
12	Jangouk,2017 [10]	68yr.	M	Subcutaneous Heparin injection	hematochezia	No	24 h	Colonoscopy	Not reported	No	No	Observation	No
13	Mirza,2019 [28]	4 days	M	Suspected necrotizing enterocolitis.	Intestinal obstruction, hematochezia, Pallor, abdominal pain.	No	–	Intraoperative	Not reported	No	No	Right hemicolectomy	Whole body edema, thrombocytopenia
14	Vecchio,2019 [3]	48 yr.	M	Spontaneous(idiopathic).	Fever, abdominal pain, intestinal obstruction, mass in the right quadrants.	Yes	–	Abdominal CT scan	Not reported	yes	yes	Right hemicolectomy	Pneumonia, Pleural effusion
15	Present study, (Alzeerelhouseini)	8 yr.	M	Falling down	Fever, anorexia, nausea and vomiting, RLQ pain, tenderness and rebound tenderness.	Yes	Six weeks	Intraoperative	10 × 8	No	No	Hematoma Evacuation	No

**Table 2:** Characteristics of all published cases (15 cases) of intramural cecal hematoma.

*Variable*	*Value*
***Average age of presentation***	*41 yr. Range (4 day – 86 yr.)*
***Male : female ratio***	*6.5 : 1*
***Causes***	
*Trauma*	*8/15 (*53 %*)*
*Colonoscopy*	*3/15 (*20 %*) {all of them were on anticoagulant thereby*}
*Hemophilia A*	*1/15 (*6.6 %*)*
*Heparin injection*	*1/15 (*6.6 %*)*
*Spontaneous(idiopathic)*	*1/15 (*6.6 %*)*
***Clinical Presentation***	
*Abdominal pain*	*13/15 (8*6.6 %*)*
*Abdominal mass*	*6/15 (*40 %*)*
*Intestinal obstruction*	*7/15 (4*6.6 %*)*
***leukocytosis***	*6/12 (*50 %*)*
***Diagnosis***	
*Abdominal CT scan*	*7/15 (4*6.6 %*)*
*Intraoperative*	*6/15 (*40 %*)*
*Colonoscopy*	*2/15 (*13.3 %*)*
***Hemoperitoneum***	*7/13 (*53.8 %*)*
***Ascending colon extension***	*9/14 (*64 %*)*
***Treatment***	
Observational	*4/15 (2*6.6 %*)*
Surgical	*11/15 (*73.3 %*)*
*Right hemicolectomy*	*9/11 (*82 %*)*
*Hematoma Evacuation*	*2/11 (*18 %*)*

The clinical presentation mainly depends on the location of the hematoma with clinical picture vary from abdominal pain to symptoms of intestinal obstruction which is the most frequent clinical picture of intestinal hematoma at the time of diagnosis [16,17]. Progression of symptoms and subsequent development of intestinal obstruction may be related to the continuous bleeding itself or it may be secondary to an osmotic effect that draws fluids from the surrounding structures [3]. Physical examination may reveal localized or diffused abdominal tenderness and peritoneal irritation suggestive of complications such as necrosis, perforation, or hemoperitoneum [2].

Laboratory tests may reveal anemia and leukocytosis. Abba et al. observed leukocytosis in 13 out of 13 patients observed with intestinal hematoma [15]. leukocytosis is probably due to the hemorrhagic disruption of the intestinal wall, with intramural and/or peritoneal diffusion of intestinal bacteria with subsequent infections [3].

According to our study, abdominal pain presented in 87 %, intestinal obstruction occurred in 47 %, and leukocytosis was observed in 50 % of cecal hematoma cases (Table 2). Moreover, all reported cases of ICH presented acutely in less than 24 h from primary insult; however, our patient presented to us with a chronic manifestation of this condition (since he came to us six weeks after he suffered the trauma) making him the first case of ICH with delayed presentation.

The diagnosis of intramural hematoma of the colon is a clinical challenge, because signs and symptoms are not specific and clinical suspicion is crucial [16]. In the pre-CT era, cecal hematoma could only be diagnosed intraoperatively [6]. Nowadays, CT scan is the gold standard for diagnosis with sensitivity is nearly 100 % [7,13,16,18]. Suggestive images of circumferential wall thickening, intramural hyperdensity, luminal narrowing, intestinal obstruction, and hyperdense ascites [16]. Abdominal ultrasound is also a useful tool for the early awareness of such lesions and it plays a great role in the follow-up evaluation of the size of the hematoma [6]. Also, Colonoscopy may be useful to show hematoma signs like “blue and roundish formations” in the submucosal layer [19]. A plain abdominal x-ray reveals only typical patterns of colonic obstruction or perforation if present [16]. However, when abdominal trauma or anticoagulant therapy is not present in the clinical history of the patient, radiologic imaging might be misleading in the diagnosis, especially when large bowel is the site of hematoma [20,21].

It should be noted that diagnosis is even more difficult to achieve in the emergency setting. In this situation, explorative laparotomy remains strongly indicated not only for diagnosis but also for treatment [3]. Our patient presented with right lower quadrant pain, fever, anorexia, nausea, and vomiting with localized tenderness and positive rebound tenderness in the right iliac fossa in association with leukocytosis which made acute appendicitis highly suspected, so urgent surgery was performed and the diagnosis of cecal hematoma was intraoperative.

The optimal management for ICH mainly depends on different etiologies and the patient’s general condition [2]. In hemodynamically stable patients especially when anticoagulant therapy or bleeding diathesis is the cause, conservative treatment is usually the preferred option which includes anticoagulant discontinuation, total parenteral nutrition, intravenous hydration, and careful observation [7,12,14,22,23]. Discontinuation of anticoagulant therapy might solve intestinal hematomas in 30 % of cases [17]. However, Surgery still may have a role in the treatment of ICH in some serious situations, especially in case of hemodynamic instability, doubtful diagnosis, failure of conservative therapy, or in presence of complications like generalized peritonitis, intestinal obstruction, or the presence of intractable bleeding and hemoperitoneum [2,3,18,24].

ICH has a relatively high risk for spontaneous rupture causing hemoperitoneum, this is mainly due to cecum intermediate disposition between the free ileum and the retroperitoneal right colon that allows intramural hematomas to expand and rupture into the peritoneal cavity. Therefore, cecal hematoma must be monitored closely because emergent right hemicolectomy has been required in all reported patients complicated by hemoperitoneum [1].

Moreover, colonic hematomas resulting from blunt injuries often require surgical intervention, because the patients are in danger of serious complications such as sepsis and abscess [25] and Prompt surgery is crucial for preventing such complications and improving the outcomes [2]. The type of operation is controversial, involving a choice between resection and evacuation of the intramural hematoma [6]. However, very few cases have been treated by evacuation and only when mucosal perforation was absent [6,19]. According to our literature, all cases of traumatic ICH have been treated by surgical intervention with right hemicolectomy is the most commonly used.

In our case, the patient was hemodynamically stable and the hematoma was not expandable or pulsatile which contains dark, black blood with many clots indicating old injury. Also, perforation, hemoperitoneum, obstruction, or extension to the ascending colon was not found. So hematoma evacuation was done and after ensuring that no oozing occurred after the evacuation, the decision to preserve the colon without resection was performed. Stretton in 1920 reported the first case of ICH treated by evacuation with limited data about the outcome [6,26]. However, our case is the second treated by evacuation which showed excellent outcome during two years follow up.

## 4 Conclusion

Intramural cecal hematoma is a strongly rare condition that could be presented with an appendicitis-like picture, so it should be considered in the differential diagnosis of acute appendicitis mainly when a history of trauma is found. Most cases of cecal hematoma presented acutely after primary insult. However, a delayed presentation can also occur. So follow-up after blunt abdominal trauma is recommended.

Conservative treatment is the first choice. However, surgery still has a great role in case of traumatic cecal hematomas, the uncertainty of diagnosis, failure of conservative management, or when complications such as intractable bleeding, perforation, or obstruction are present.

## Declaration of Competing Interest

There is no conflict of interest.

## Sources of funding

No funding or grant support.

## Ethical approval

The study is exempt from ethical approval in our institution.

## Consent

Written informed consent was obtained from the patient's parents for publication of this case report and accompanying images. A copy of the written consent is available for review by the Editor-in-Chief of this journal on request

## Author contribution

Study concept or design: Osama Y. Aljabarein, Hussam I. A. Alzeerelhouseini.

Data collection and data analysis: Hussam I. A. Alzeerelhouseini.

Writing the manuscript: Hussam I. A. Alzeerelhouseini, Yousef S. Abuzneid.

Review & editing the manuscript: Hussam I. A. Alzeerelhouseini.

## Registration of research studies

Not applicable.

## Guarantor

Dr. Hussam I. A. Alzeerelhouseini.

## Provenance and peer review

Not commissioned, externally peer-reviewed.
